# Supplementary material for: Identification of photoperiod-induced specific miRNAs in the adrenal glands of Sunite sheep (Ovis aries)
Source: Front Vet Sci. 2022 Jul 22;9:888207. doi: 10.3389/fvets.2022.888207 (PMC9354845; doi:10.3389/fvets.2022.888207)
Supplement: Supplementary file 1 [file Data_Sheet_1.ZIP › Supplementary file/miRDeep2 file.zip!/miRDeep2 file/Know_Structure/LP42A3/oar-mir-17.pdf]

miRBase precursor : oar-mir-17
Total read count : 3536
oar-miR-17-5p read count : 3490
remaining reads : 46

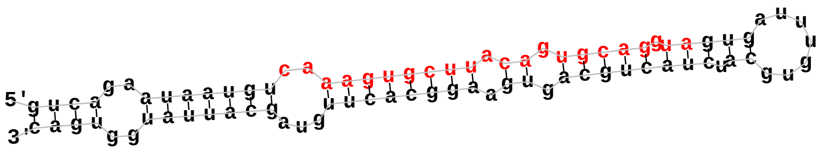

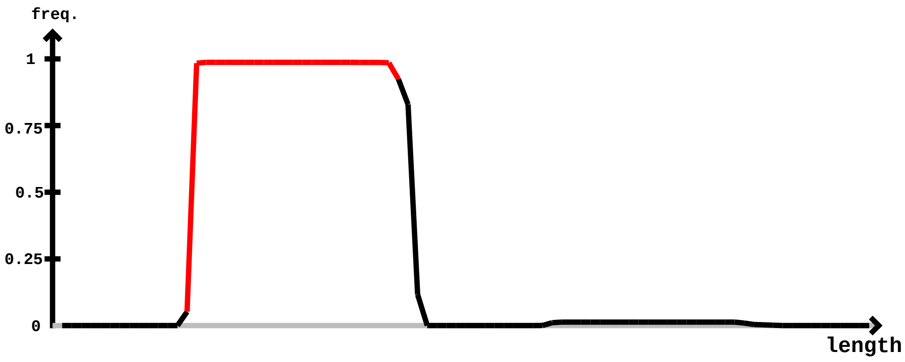


```
                 oar-miR-17-5p
5'- gucagaauaaugucaaagugcuuacagugcagguagugauuugugcaucuacugcagugaaggcacuuguagcauuauggugac  -3'    exp
    ((((..(((((((..((((((((.((.(((((.(((((.......)).)))))))).)).))))))))...)))))))..))))    reads      mm         sample
    ............ucaaagugcuuacagugcagg...................................................    3          0          seq
    ............ucaaagugcuuacagugcaggu..................................................    28         0          seq
    ............ucaaagugcuuacagugcaggua.................................................    54         0          seq
    ............ucaaagugcuuacagugcagguag................................................    94         0          seq
    ............ucaaagugcuuacagugcagguagu...............................................    4          0          seq
    .............caaagugcuuacagugc......................................................    1          0          seq
    .............caaagugcuuacagugcagg...................................................    2          0          seq
    .............caaagugcuCacagugcaggu..................................................    1          1          seq
    .............caaagugcuuacagugcaggu..................................................    186        0          seq
    .............caaagugcuuacagugcaggua.................................................    274        0          seq
    .............caaagugcuCacagugcaggua.................................................    5          1          seq
    .............caaagugcuCacagugcagguag................................................    23         1          seq
    .............caaagugcuuacagugcagguag................................................    2398       0          seq
    .............caaagugcuuacagugcagguagu...............................................    406        0          seq
    ..............aaagugcuuacagugcaggu..................................................    1          0          seq
    ..............aaagugcuCacagugcaggu..................................................    1          1          seq
    ..............aaagugcuuacagugcaggua.................................................    1          0          seq
    ..............aaagugcuuacagugcagguag................................................    6          0          seq
    ..............aaagugcuuaUagugcagguag................................................    1          1          seq
    ..............aaagugcuuaUagugcagguagu...............................................    1          1          seq
    .................................................uacugcagugaaggcacuuguag............    1          0          seq
    ..................................................acugcagugaaggcacuugu..............    12         0          seq
    ..................................................acugcagugaaggcacuugua.............    19         0          seq
    ..................................................acugcagugaaggcacuuguag............    3          0          seq
    ..................................................acugcagugaaggcacuuguagca..........    3          0          seq
    ...................................................cugcagugaaggcacuugu..............    1          0          seq
    ...................................................cugcagugaaggcacuuguagc...........    5          0          seq
    ...................................................cugcagugaaggcacuuguagca..........    2          0          seq
```
